# Attitudes toward cost-conscious care among U.S. physicians and medical students: analysis of national cross-sectional survey data by age and stage of training

**DOI:** 10.1186/s12909-018-1388-7

**Published:** 2018-11-22

**Authors:** Andrea N. Leep Hunderfund, Liselotte N. Dyrbye, Stephanie R. Starr, Jay Mandrekar, Jon C. Tilburt, Paul George, Elizabeth G. Baxley, Jed D. Gonzalo, Christopher Moriates, Susan D. Goold, Patricia A. Carney, Bonnie M. Miller, Sara J. Grethlein, Tonya L. Fancher, Matthew K. Wynia, Darcy A. Reed

**Affiliations:** 1 Neurology, Mayo Clinic, 200 First Street SW, Rochester, MN 55905 USA; 2 Medical education and medicine, Mayo Clinic, 200 First Street SW, Rochester, MN 55905 USA; 3 Science of Health Care Delivery Education, Mayo Clinic School of Medicine, Mayo Clinic, 200 First Street SW, Rochester, MN 55905 USA; 4 Biostatistics and Neurology, Mayo Clinic, 200 First Street SW, Rochester, MN 55905 USA; 5 Biomedical ethics, Mayo Clinic, 200 First Street SW, Rochester, MN 55905 USA; 6 Family medicine and medical science, Warren Alpert Medical School, Brown University, 222 Richmond Street, Providence, RI 02903 USA; 7 Family medicine, Brody School of Medicine, East Carolina University, 600 Moye Blvd, Greenville, NC 27834 USA; 8 Medicine and public health sciences and associate dean for health systems education, Pennsylvania State University College of Medicine, 500 University Drive, Hershey, PA 17033 USA; 9 Division of Hospital Medicine, and director, Caring Wisely Program, University of California San Francisco, San Francisco, California, USA; 10 Dell Medical School at the University of Texas at Austin, 1501 Red River Road, Health Learning Building, Austin, TX 78701 USA; 11 Internal medicine and health management, Center for Bioethics and Social Sciences in Medicine, University of Michigan, 500 South State Street, Ann Arbor, MI 48109 USA; 12 Family medicine and of public health and preventative medicine, Oregon Health & Science University, 3181 SW Sam Jackson Park Rd, Portland, OR 97239 USA; 13 Medical education and administration, professor of clinical surgery, associate vice chancellor for health affairs, and senior associate dean for health sciences education, Vanderbilt University, 2201 West End Ave, Nashville, TN 37235 USA; 14 Clinical medicine, Department of Medicine, Indiana University School of Medicine, 340 W 10th St 6200, Indianapolis, IN 46202 USA; 15 Division of General Medicine, Medicine and associate dean for workforce innovation and community engagement, University of California Davis School of Medicine, 4610 X Street, Sacramento, CA 95817 USA; 16 Internal medicine, Center for Bioethics and Humanities at the University of Colorado Denver, 1250 14th Street, Denver, CO 80204 USA; 17 Medical education and medicine, Mayo Clinic School of Medicine, Mayo Clinic, 200 First Street SW, Rochester, MN 55905 USA

**Keywords:** Cost-conscious care, High value cost-conscious care, High value care, Value-based health care, Healthcare costs, Health care costs, Undergraduate medical education, National survey, Cohort effect, Generational differences

## Abstract

**Background:**

The success of initiatives intended to increase the value of health care depends, in part, on the degree to which cost-conscious care is endorsed by current and future physicians. This study aimed to first analyze attitudes of U.S. physicians by age and then compare the attitudes of physicians and medical students.

**Methods:**

A paper survey was mailed in mid-2012 to 3897 practicing physicians randomly selected from the American Medical Association Masterfile. An electronic survey was sent in early 2015 to all 5,992 students at 10 U.S. medical schools. Survey items measured attitudes toward cost-conscious care and perceived responsibility for reducing healthcare costs. Physician responses were first compared across age groups (30–40 years, 41–50 years, 51–60 years, and > 60 years) and then compared to student responses using Chi square tests and logistic regression analyses (controlling for sex).

**Results:**

A total of 2,556 physicians (65%) and 3395 students (57%) responded. Physician attitudes generally did not differ by age, but differed significantly from those of students. Specifically, students were more likely than physicians to agree that cost to society should be important in treatment decisions (*p* < 0.001) and that physicians should sometimes deny beneficial but costly services (*p* < 0.001). Students were less likely to agree that it is unfair to ask physicians to be cost-conscious while prioritizing patient welfare (*p* < 0.001). Compared to physicians, students assigned more responsibility for reducing healthcare costs to hospitals and health systems (*p* < 0.001) and less responsibility to lawyers (*p* < 0.001) and patients (*p* < 0.001). Nearly all significant differences persisted after controlling for sex and when only the youngest physicians were compared to students.

**Conclusions:**

Physician attitudes toward cost-conscious care are similar across age groups. However, physician attitudes differ significantly from medical students, even among the youngest physicians most proximate to students in age. Medical student responses suggest they are more accepting of cost-conscious care than physicians and attribute more responsibility for reducing costs to organizations and systems rather than individuals. This may be due to the combined effects of generational differences, new medical school curricula, students’ relative inexperience providing cost-conscious care within complex healthcare systems, and the rapidly evolving U.S. healthcare system.

## Background

Healthcare costs are growing at unsustainable rates, especially in the United States (U.S.) where spending on healthcare reached $3.2 trillion in 2015. [1, 2] This accounts for more than 17% of the U.S. economy, [2] with additional increases projected due to expanded insurance coverage and an aging population. [3] Increasing costs threaten the financial sustainability of the U.S. healthcare system, [1, 4] prevent allocation of resources to other important societal needs, [1] and often burden patients with significant out-of-pocket expenses. [5, 6] Data like these have galvanized the high-value, cost-conscious care movement [7] with numerous calls to reduce waste, [8] consider the financial impact of care decisions, [9, 10] increase cost transparency, [11, 12] reimburse physicians for value rather than volume, [13] and equip medical trainees with the knowledge, attitudes, and skills they will need to practice cost-conscious care. [7, 14]

Physician attitudes are a key driver of overuse (the provision of healthcare services for which there is no medical basis or for which harms equal or exceed benefits), [15–17] which increases costs and undermines the quality of care. [8, 16] This is evidenced by multiple studies demonstrating that physician attitudes and beliefs are associated with their utilization of healthcare services. [18–21] Data further suggest that younger, less experienced physicians tend to provide more expensive care than older, more experienced physicians. [22] This could in part reflect age-related differences in attitudes toward cost-conscious care, which is of particular importance given the aging physician workforce. [23] However, the degree to which physician attitudes vary by age is unknown.

A better understanding of physician attitudes toward cost-conscious care is critical, as the success of resulting cost-conscious care initiatives (such as the “Choosing Wisely” campaign [24]) depends, in part, on the extent to which they are embraced and endorsed by current and future physicians. The attitudes and behaviors of practicing physicians also play a large role in shaping the learning environment medical students encounter with respect to cost-conscious care. [25] Students, in turn, must reconcile their attitudes toward cost-conscious care with those of their supervisors as part of their professional identity formation process. [26] Prior studies have examined attitudes toward cost-conscious care among U.S. physicians [27–29] and medical students. [25] However, only limited attempts have been made to compare attitudes among these groups. [30]

In this study, we aimed to address these gaps by analyzing the attitudes of U.S. physicians by age and then comparing physician attitudes with those of medical students. The results can inform cost containment efforts targeting physicians in practice, as well as initiatives in medical education intended to promote cost-conscious care among faculty and students.

## Methods

### Study design

We conducted a secondary analysis of data from the Physicians, Healthcare Costs, and Society survey administered in mid-2012 [28, 31, 32] and the Medical Student Attitudes Toward and Experiences with Cost-Conscious Care survey administered in early 2015. [25]

### Ethics and consent

The Mayo Clinic Institutional Review Board deemed the Physicians, Healthcare Costs, and Society survey study exempt. The institutional review boards of each participating school exempted or approved the Medical Student Attitudes Toward and Experiences with Cost-Conscious Care survey study. Consent for both was implied upon survey completion.

### Participants and data collection

#### Practicing physician sample

As previously described, [28] three of us (JCT, MKW, SDG) mailed a self-administered paper survey between May 1 and July 31, 2012 to 3897 practicing physicians randomly selected from the AMA Physician Masterfile. The AMA Physician Masterfile is the most comprehensive listing of U.S. physicians and is independent of AMA membership. [33] This sample included physicians from all specialties except those whose primary specialty was listed as administration only. The first survey mailing included a $20 bill. Two additional mailings were sent to non-respondents at six-week intervals.

#### Medical student sample

As previously reported, [25] we surveyed all 5992 students at 10 U.S. medical schools between January 1, 2015 and March 31, 2015. Participating schools included the Warren Alpert Medical School of Brown University; Brody School of Medicine at East Carolina University; University of California, San Francisco School of Medicine; University of California, Davis School of Medicine; Indiana University School of Medicine, Mayo Medical School; University of Michigan Medical School; Oregon Health & Science University School of Medicine; Pennsylvania State University College of Medicine; and Vanderbilt University School of Medicine. These schools were recruited through the American Medical Association (AMA) Accelerating Change in Medical Education consortium [34] and vary with respect to geographic region, public/private status, class size, and mission. Medical students received an email invitation to participate in the study along with a link to the survey. We sent up to three reminders to non-respondents. Participation was voluntary, and all responses were anonymous. Students at nine schools were offered an opportunity to enter a lottery to win a $250 cash card as an incentive for participation; one school did not allow an incentive.

### Study measures

The physician and medical student surveys both contained items measuring attitudes toward cost-conscious care and perceived responsibility for reducing healthcare costs. As previously described, survey items were pilot tested with both physicians [28] and medical students [25] prior to administration with revisions based on their feedback. Items assessing respondents’ attitudes toward cost-conscious care were derived from peer-reviewed publications, including a Cost-Consciousness Scale (e.g., “Trying to contain costs is the responsibility of every physician”), [27] the Agreement with Rationing Scale (e.g., “Cost to society should be important in physician decisions to use or not to use an intervention”), [35] and a Stewardship Scale developed by the American Medical Association’s Institute for Ethics (e.g., “Physicians should try not to think about the cost to the healthcare system when making treatment decisions” [reverse scored]). [28] Additional items were developed based on a literature review and focus groups with physicians (e.g., “Physicians should be solely devoted to individual patients’ best interests, even if that is expensive”), as previously described. [28] These peer-reviewed publications include reliability and validity evidence supporting the use of these items to measure physician attitudes toward cost-conscious care. [27, 28, 35] Some items were modified slightly to make them appropriate for medical students (e.g., changing “I should” to “physicians should”). Respondents were asked to indicate their extent of agreement on a four-point Likert scale (1 = strongly disagree, 2 = moderately disagree, 3 = moderately agree, 4 = strongly agree).

Nine items asked respondents to indicate their views on the perceived responsibility of different stakeholder groups (health insurance companies, hospitals and health systems, pharmaceutical and device manufacturers, government, physician professional societies, employers, trial lawyers, patients, and individual practicing physicians) for reducing healthcare costs using a three-point Likert scale (1 = no responsibility, 2 = some responsibility, 3 = major responsibility). These items were developed for the physician survey based on a literature review and focus groups with physicians. [28]

### Data analysis

Response rates were reported using the American Association for Public Opinion Research RR2 response rate definition. [36] Items measuring attitudes toward cost-conscious care were dichotomized as moderately/strongly agree vs. moderately/strongly disagree. Items measuring perceived responsibility for reducing healthcare costs were dichotomized as major responsibility vs. some/no responsibility to approximate the mid-point of the distribution of survey responses to these items. Descriptive summary statistics were reported as frequencies with percentages. Pearson chi square tests were used to evaluate unadjusted differences among proportions. Multivariate logistic regression models were used to evaluate differences between the attitudes of physicians of different age and medical students controlling for sex. For two items, physicians were asked what they *actually* do whereas medical students were asked what physicians *should* do. Thus, although they measured similar constructs, direct statistical comparisons of student and physician responses to these two items were not performed. The Bonferroni method was used to correct for multiple comparisons by dividing our original α of 0.05 by the number of analyses we performed (*n* = 120), rounded to three decimal points. In this manner, we specified significance at *p* < 0.001 using two-sided testing. Analyses were performed using SAS version 9.3 (SAS Inc., Cary NC).

## Results

As previously reported, 2556/3897 (65%) practicing physicians [28] and 3395/5992 (57%) medical students responded. [25] The sex and age of physician and medical student respondents were similar to those of the overall samples [14, 25, 28] and to U.S. physicians [37] and medical students [38] in general. Specifically, physician respondents were 70% male (1784/2556) compared to 70% (2742/3897) of the overall sample, and student respondents were 49% male (1428/3395) compared to 51% (3049/5992) of the overall sample. Fourteen percent (350/2556) of physician respondents were age 30 to 40 years, 33% (833/2556) were age 41 to 50 years, 38% (962/2556) were age 51 to 60 years, and 16% (411/2556) were over 60 years at the time of survey administration, whereas 90% (2657/2958) of medical student respondents were 30 years old or younger.

### Attitudes toward cost-conscious care

As shown in Table 1, physician attitudes toward cost-conscious care differed by age for only one of the nine survey items. Specifically, younger physicians were more likely to moderately or strongly agree that doctors are too busy to worry about the costs of tests and procedures (*p* < 0.001). Otherwise, the attitudes of physicians were similar across age groups.

**Table 1:** Attitudes of U.S. Practicing Physicians and Medical Students toward Cost-Conscious Care

Survey Item^a^	Moderately or Strongly Agree, n (%)^b^								
	Practicing physicians (*n* = 2556)						Medical students (n = 3395)		
	Age > 60 years (*n* = 411)	Age 51–60 years (*n* = 962)	Age 41–50 years (*n* = 833)	Age 30–40 years (*n* = 350)	*p*-value		All years^e^(*n* = 3395)	*p*-value	
					Unadjusted^c^	Adjusted for sex^d^		Unadjusted^c^	Adjusted for sex^d^
Doctors are too busy to worry about the costs of tests and procedures.	86/386 (22)	217/921 (24)	238/802 (30)	112/342 (33)	< 0.001	< 0.001	995/2947 (34)	< 0.001	< 0.001
Trying to contain costs is the responsibility of every physician.	334/383 (87)	800/919 (87)	659/798 (83)	286/342 (84)	0.03	0.06	2640/2932 (90)	< 0.001	< 0.001
Physicians should take a more prominent role in limiting use of unnecessary tests.	344/381 (90)	825/922 (89)	695/797 (87)	298/342 (87)	0.26	0.30	2896/3003 (96)	< 0.001	< 0.001
Cost to society should be important in physician decisions to use or not to use an intervention.	218/382 (57)	492/919 (54)	400/796 (50)	197/342 (58)	0.05	0.06	2062/2951 (70)	< 0.001	< 0.001
Physicians should sometimes deny beneficial but costly services to certain patients because resources should go to other patients who need them more.	60/381 (16)	140/913 (15)	119/794 (15)	47/340 (14)	0.90	0.03	1024/2987 (34)	< 0.001	< 0.001
It is unfair to ask physicians to be cost-conscious and still keep the welfare of their patients foremost in their minds.	147/383 (38)	359/919 (39)	359/794 (45)	158/343 (46)	0.01	0.02	887/2950 (30)	< 0.001	< 0.001
Practicing cost-conscious care will undermine patients’ trust in physicians.^f^	101/389 (26)	255/913 (28)	246/780 (32)	102/327 (31)	0.15	0.01	482/2931 (16)	< 0.001	< 0.001
Physicians should be aware of the costs of the tests or treatments they recommend.	297/384 (77)	719/921 (78)	608/798 (76)	239/434 (70)	0.02	0.003	2920/3000 (97)	N/A^g^	N/A^g^
Physicians should try not to think about the cost to the health care system when making treatment decisions.	156/386 (40)	359/920 (39)	354/800 (44)	143/343 (42)	0.17	0.27	652/2997 (22)	N/A^g^	N/A^g^
Physicians should be solely devoted to individual patients’ best interests, even if that is expensive.	299/383 (78)	708/918 (77)	623/797 (78)	272/340 (80)	0.75	0.78	2265/3005 (75)	0.17	0.05
The cost of a test or medication is only important if the patient has to pay for it out of pocket.	63/385 (16)	126/921 (14)	130/800 (16)	68/343 (20)	0.06	0.05	399/2951 (14)	0.009	0.002

^a^Survey items listed as they appeared in the medical student survey. Unless otherwise indicated, physicians (surveyed in mid-2012) and medical students (surveyed in early 2015) were asked to indicate their extent of agreement on a four-point Likert scale (1 = strongly disagree, 2 = moderately disagree, 3 = moderately agree, 4 = strongly agree). Data for medical students are previously published. [17]

^b^Percentages not all based on a denominator of 350 (for physicians age 30–40 years), 833 (for physicians age 41–50 years), 962 (for physicians age 51–60 years), 411 (for physicians age > 60 years), or 3395 (for students) because of missing responses to some survey items

^c^Pearson Chi square test; *p*-values < 0.001 considered statistically significant

^d^Multivariate logistic regression controlling for sex; p-values < 0.001 considered statistically significant

^e^Most medical students were age 30 or younger (2657/2958, 90%)

^f^Physicians were asked to indicate their extent of agreement with this item by checking a box (checked = agree, unchecked = disagree) as part of a “check all that apply” question; the denominator for this item represents the number of respondents who checked any box associated with this question

^g^For these items, medical students were asked what physicians *should* do whereas physicians were asked what they *actually* do. Thus, although survey items measured similar constructs, direct statistical comparisons of student and physician responses were not performed

However, when the attitudes of physicians were compared with those of medical students, there were several significant differences (Table 1). Medical students were more likely to moderately or strongly agree that cost to society should be important in physician decisions to use or not to use an intervention (*p* < 0.001) and to agree with the concept of rationing (i.e., that physicians should sometimes deny beneficial but costly services to certain patients because resources should go to other patients who need them more; *p* < 0.001). Students were also more likely to agree that trying to contain costs is the responsibility of every physician (*p* < 0.001) and that physicians should take a more prominent role in limiting the use of unnecessary tests (*p* < 0.001), though rates of agreement with these statements were high across all groups. Conversely, students were less likely than physicians to moderately or strongly agree that it is unfair to ask physicians to be cost-conscious and still keep the welfare of their patients foremost in their minds (*p* < 0.001) and that practicing cost-conscious care will undermine patients’ trust in physicians (*p* < 0.001).

Nearly all medical students agreed that physicians should be aware of the costs of the tests or treatments they recommend (2920/3000, 97%), yet only 76% (1863/2446) of physicians (across all ages) indicated that they are actually aware of these costs. Only 22% (652/2997) of students agreed that physicians should try not to think about the cost to the healthcare system when making treatment decisions, whereas 41% (1012/2449) of physicians (across all ages) agreed that they actually do this.

Despite these differences, the majority of both students and physicians of all ages agreed that physicians should be solely devoted to individual patients’ best interests even if that is expensive and disagreed that the cost of a test or medication is only important if the patient has to pay for it out of pocket.

### Perceived responsibility for reducing costs of care

As shown in Table 2, younger physicians attributed more responsibility for reducing healthcare costs to health insurance companies than older physicians (*p* < 0.001). The responsibility attributed to other entities was otherwise similar across physician age groups.

**Table 2:** Perceived Responsibility for Reducing Healthcare Costs among U.S. Practicing Physicians and Medical Students^a^

Entities with potential responsibility to reduce costs of health care	Major responsibility, n (%)^b^								
	Practicing physicians (*n* = 2556)						Medical students (*n* = 3395)		
	Age > 60 years (*n* = 411)	Age 51–60 years (*n* = 962)	Age 41–50 years (*n* = 833)	Age 30–40 years (*n* = 350)	*p*-value		All years^e^ (*n* = 3395)	*p*-value	
					Unadjusted^c^	Adjusted for sex^d^		Unadjusted^c^	Adjusted for sex^d^
Health insurance companies	224/385 (58)	494/920 (54)	499/804 (62)	222/337 (66)	< 0.001	< 0.001	2166/2933 (74)	< 0.001	< 0.001
Hospitals and health systems	226/383 (59)	528/917 (58)	437/803 (54)	182/336 (54)	0.32	0.44	2297/2930 (78)	< 0.001	< 0.001
Pharmaceutical and device manufacturers	217/385 (56)	506/921 (55)	458/803 (57)	196/336 (58)	0.70	0.003	1971/2932 (67)	< 0.001	< 0.001
Government	186/382 (49)	400/919 (44)	333/803 (41)	154/336 (46)	0.11	0.14	1886/2932 (64)	< 0.001	< 0.001
Physician professional societies	119/381 (31)	256/917 (28)	207/801 (26)	85/334 (25)	0.21	0.28	1218/2933 (42)	< 0.001	< 0.001
Employers	81/380 (21)	177/917 (19)	143/798 (18)	56/334 (17)	0.39	0.45	860/2925 (29)	< 0.001	< 0.001
Trial lawyers	220/383 (57)	557/914 (61)	486/803 (61)	187/333 (56)	0.34	0.50	954/2918 (33)	< 0.001	< 0.001
Patients	203/381 (53)	477/920 (52)	413/803 (51)	172/335 (51)	0.94	0.03	706/2929 (24)	< 0.001	< 0.001
Individual practicing physicians	153/379 (40)	359/919 (39)	257/804 (32)	120/336 (36)	0.007	< 0.001	1171/2933 (40)	0.001	< 0.001

^a^Physicians (surveyed in mid-2012) and medical students (surveyed in early 2015) and were asked to rate the degree of responsibility (if any) each entity should have in reducing healthcare costs using a three-point scale (1 = no responsibility, 2 = some responsibility, 3 = major responsibility)

^b^Percentages not all based on a denominator of 350 (for physicians age 30–40 years), 833 (for physicians age 41–50 years), 962 (for physicians age 51–60 years), 411 (for physicians age > 60 years), or 3395 (for students) because of missing responses to some survey items

^c^Pearson Chi square test; *p*-values < 0.001 considered statistically significant

^d^Multivariate logistic regression controlling for sex; *p*-values < 0.001 considered statistically significant

^e^Most medical students were age 30 or younger (2657/2958, 90%)

However, when physician perceptions were compared with those of medical students, there were again a number of significant differences (Table 2). Specifically, students attributed more responsibility to health insurance companies, hospitals and health systems, pharmaceutical and device manufacturers, government, physician professional societies, and employers than physicians (all *p* < 0.001) and less responsibility to trial lawyers and patients (both *p* < 0.001). Despite these differences, a similar proportion of students and physicians (across all age groups) agreed that individual practicing physicians have a major responsibility for reducing healthcare costs.

### Sensitivity analyses

As the proportion of male respondents was overall much higher among physician respondents than student respondents (1784/2556, 70% vs. 1428/3395, 49%; *p* < 0.001), we repeated our analyses controlling for sex. As shown in Tables 1 and 2, significant differences between comparison groups persisted for all but one survey item (fewer physicians age 41 to 50 attributed major responsibility for reducing healthcare costs to individual practicing physicians compared to the other groups, *p* < 0.001 after controlling for sex).

Because the youngest group of physicians (those age 30–40 years) were most proximate to medical students in age and stage of training and most similar to students with respect to sex (190/350, 54% vs. 1428/3395, 49%; *p* = 0.04), we repeated statistical comparisons including only these two groups. As shown in Table 3, the attitudes of medical students still differed significantly from those of the youngest physicians for six of the nine survey items (all *p* < 0.001), and all but one of these differences persisted after controlling for sex. Students also still attributed more responsibility to hospitals and health systems, government, physician professional societies, and employers (all *p* < 0.001) and less responsibility to trial lawyers and patients (both *p* < 0.001) than the youngest physicians (Table 4). These differences also persisted after controlling for sex.

**Table 3:** Attitudes of U.S. Practicing Physicians (Age 30–40) and Medical Students toward Cost-Conscious Care

Survey Item^a^	Moderately or Strongly Agree, n (%)^b^			
	Practicing physicians	Medical students	*p*-value	
	Age 30–40 years (*n* = 350)	All years^c^ (*n* = 3395)	Unadjusted^d^	Adjusted for sex^e^
Doctors are too busy to worry about the costs of tests and procedures.	112/342 (33)	995/2947 (34)	0.71	0.76
Trying to contain costs is the responsibility of every physician.	286/342 (84)	2640/2932 (90)	< 0.001	0.001
Physicians should take a more prominent role in limiting use of unnecessary tests.	298/342 (87)	2896/3003 (96)	< 0.001	< 0.001
Cost to society should be important in physician decisions to use or not to use an intervention.	197/342 (58)	2062/2951 (70)	< 0.001	< 0.001
Physicians should sometimes deny beneficial but costly services to certain patients because resources should go to other patients who need them more.	47/340 (14)	1024/2987 (34)	< 0.001	< 0.001
It is unfair to ask physicians to be cost-conscious and still keep the welfare of their patients foremost in their minds.	158/343 (46)	887/2950 (30)	< 0.001	< 0.001
Practicing cost-conscious care will undermine patients’ trust in physicians.^f^	102/327 (31)	482/2931 (16)	< 0.001	< 0.001
Physicians should be aware of the costs of the tests or treatments they recommend.	239/434 (70)	2920/3000 (97)	N/A^g^	N/A^g^
Physicians should try not to think about the cost to the health care system when making treatment decisions.	143/343 (42)	652/2997 (22)	N/A^g^	N/A^g^
Physicians should be solely devoted to individual patients’ best interests, even if that is expensive.	272/340 (80)	2265/3005 (75)	0.06	0.03
The cost of a test or medication is only important if the patient has to pay for it out of pocket.	68/343 (20)	399/2951 (14)	0.002	0.001

^a^Survey items listed as they appeared in the medical student survey. Unless otherwise indicated, physicians (surveyed in mid-2012) and medical students (surveyed in early 2015) were asked to indicate their extent of agreement on a four-point Likert scale (1 = strongly disagree, 2 = moderately disagree, 3 = moderately agree, 4 = strongly agree). Data for medical students are previously published. [17]

^b^Percentages not all based on a denominator of 350 (for physicians age 30–40 years) or 3395 (for students) because of missing responses to some survey items

^c^Most medical students were age 30 or younger (2657/2958, 90%)

^c^Pearson Chi square test; *p*-values < 0.001 considered statistically significant

^d^Multivariate logistic regression controlling for sex; p-values < 0.001 considered statistically significant

^f^Physicians were asked to indicate their extent of agreement with this item by checking a box (checked = agree, unchecked = disagree) as part of a “check all that apply” question; the denominator for this item represents the number of respondents who checked any box associated with this question

^g^For these items, medical students were asked what physicians *should* do whereas physicians were asked what they *actually* do. Thus, although survey items measured similar constructs, direct statistical comparisons of student and physician responses were not performed

**Table 4:** Perceived Responsibility for Reducing Healthcare Costs among U.S. Practicing Physicians (Age 30–40) and Medical Students^a^

Entities with potential responsibility to reduce costs of health care	Major responsibility, n (%)^b^			
	Practicing physicians	Medical students	*p*-value	
	Age 30–40 years (*n* = 350)	All years^c^ (*n* = 3395)	Unadjusted^d^	Adjusted for sex^e^
Health insurance companies	222/337 (66)	2166/2933 (74)	0.002	0.001
Hospitals and health systems	182/336 (54)	2297/2930 (78)	< 0.001	< 0.001
Pharmaceutical and device manufacturers	196/336 (58)	1971/2932 (67)	0.001	< 0.001
Government	154/336 (46)	1886/2932 (64)	< 0.001	< 0.001
Physician professional societies	85/334 (25)	1218/2933 (42)	< 0.001	< 0.001
Employers	56/334 (17)	860/2925 (29)	< 0.001	< 0.001
Trial lawyers	187/333 (56)	954/2918 (33)	< 0.001	< 0.001
Patients	172/335 (51)	706/2929 (24)	< 0.001	< 0.001
Individual practicing physicians	120/336 (36)	1171/2933 (40)	0.13	0.001

^a^Physicians (surveyed in mid-2012) and medical students (surveyed in early 2015) and were asked to rate the degree of responsibility (if any) each entity should have in reducing healthcare costs using a three-point scale (1 = no responsibility, 2 = some responsibility, 3 = major responsibility)

^b^Percentages not all based on a denominator of 350 (for physicians age 30–40 years) or 3395 (for students) because of missing responses to some survey items

^c^Most medical students were age 30 or younger (2657/2958, 90%)

^c^Pearson Chi square test; *p*-values < 0.001 considered statistically significant

^d^Multivariate logistic regression controlling for sex; *p*-values < 0.001 considered statistically significant

## Discussion

To our knowledge, this is the first large, national study to analyze age-based differences in U.S. physician attitudes toward cost-conscious care and examine differences based on stage of training (practicing physicians vs. medical students). We found that physician attitudes generally did not differ by age, suggesting that higher costs among younger physicians may be driven by other factors, such as treating patients with more expensive ailments or greater discomfort with uncertainty, [27] and that receptivity to cost containment efforts may be similar across physician age groups.

While physician attitudes toward cost-conscious care generally did not differ by age, we found a number of significant differences between physicians and medical students. When compared to physicians, students were more accepting of cost-conscious care, were more comfortable with the concept of rationing (i.e. denying beneficial but costly services to certain patients because resources should go to other patients who need them more), and attributed more responsibility for reducing costs to organizations and systems rather than individuals. Nearly all these differences persisted after controlling for sex and even when responses from only the youngest physicians (those most proximate to medical students in age and career stage) were included.

There are many potential explanations for these findings. First, the attitudes of practicing physicians and medical students may reflect a cohort effect. The physician respondents in this study were from either the Baby Boomer generation (those over age 50) or Generation X (those age 30 to 50), whereas most medical students were age 30 or younger, putting them in the Millennial generation. [39] Compared to preceding generations, Millennials have a greater affinity for teams. [40] They tend to be more assertive, globally minded, and motivated to tackle big social issues. [41, 42] Millennial students have also experienced a healthcare environment that is very different from the one many physicians experienced during their training and are entering medicine at a time of record high tuition fees and education debt. [43] This may make them more sensitive to issues of cost, as has been previously suggested. [30] These characteristics and experiences may make Millennials more systems-oriented than their predecessors and more predisposed to addressing the problem of healthcare costs.

Second, medical education has changed a great deal over the past decade, and medical student attitudes may in part reflect these changes. For example, topics such as systems-based practice, healthcare economics, and cost-conscious care are increasingly common in medical school curricula, [44, 45] and the number of joint Medical Doctor / Master of Business Administration programs in the US has grown from 6 to 65 over the past 20 years. [46] These changes are intended to better equip students to meet the needs of society, including the problem of rising healthcare costs. Conversely, practicing physicians may have received little or no formal training in such topics. Thus, these physicians may not be optimally positioned to reinforce what students are learning in their formal curriculum or equip students with the knowledge, attitudes, and skills they need to provide cost-conscious care.

Third, medical student attitudes may reflect an element of naivety, as students have yet to fully experience the realities of day-to-day practice for a physician with its accompanying pressures and complexities. [47] For example, it may be much easier for a student to agree with the concept of rationing (i.e. denying beneficial but costly services to certain patients because resources should go to other patients who need them more) when they have not yet been in the position of making such a decision or delivering such a message to patients. Practicing physicians, on the other hand, actually carry the weight of responsibility for patient care and are more familiar with their own limitations and the limitations of the healthcare system, which poses many obstacles to the practice of cost-conscious care. [48]

From this view, the attitudes of medical students may be expected to change over time as they become more engaged in and responsible for patient care. Previous data suggest medical student attitudes vary little by year in school. [25] However, we found significant differences between the attitudes of medical students and the youngest group of practicing physicians, suggesting that residency may be a particularly critical time in the physician socialization process. Indeed, prior studies have shown that practice patterns experienced during residency training are correlated with physicians’ future knowledge, [49] attitudes, [50] and behaviors [51] with respect to cost-conscious care.

Efforts to promote cost-conscious care in graduate medical education (such as the American College of Physicians High Value Care curriculum, [52] the Teaching Value and Choosing Wisely challenge, [24] and the Costs of Care Teaching Value in Healthcare Learning Network [53]) are already underway. Multifaceted training and socialization strategies that simultaneously engage practicing physicians, residents, and medical students, [54] address the systems within which they operate, [55–57] and emphasize physicians’ primary commitment to patient welfare [58] have been recommended. Research about what strategies (e.g., communication methods) and circumstances (e.g., levels of trust) facilitate or hamper cost conversations between doctors and patients should inform these efforts. Healthcare payment reform (linking reimbursement to value rather than volume), cost transparency, clinical decision support technology, and utilization reports with benchmarking to peers may also serve to promote cost-conscious care among practicing physicians. [4, 13]

If current medical students sustain their positive attitudes toward cost-conscious care, optimism regarding the future of the high-value, cost-conscious care movement may be in order. Better defining what that does and does not entail for medical professionalism will be a key pedagogical task in the coming years. In the meantime, efforts to teach cost-conscious care may be able to focus less on arguing for its importance, which the great majority of students and physicians now accept, and more on equipping current and future physicians with practical strategies for providing cost-conscious care within the complexities of heterogeneous health systems and in relation to their simultaneous role as advocates for individual patients. Medical students can further be empowered to serve as change agents in their learning environment by asking questions, challenging assumptions, including value in their case presentations, [59] and approaching the problem of healthcare costs from a systems perspective. [54] Although students identify the healthcare team hierarchy as a potential barrier, many nevertheless indicate they would be comfortable broaching the topic of high-value, cost-conscious care with their team. [54] Some schools have even formalized this role by enlisting medical students to serve as “High Value Care Officers” for their inpatient teams. [60]

The generalizability of these results is supported by strong response rates (which reduce concerns about response bias) and the inclusion of practicing physicians and medical students who are geographically distributed across the U.S. Nevertheless, this study has limitations. First, although the AMA Masterfile is the most comprehensive listing of U.S. physicians, the findings reported here may not fully reflect the opinions of all U.S. physicians. [28] Similarly, the 10 schools that participated in the medical student survey were recruited through the AMA Accelerating Change in Medical Education initiative, [34] so the responses of students from these schools may not reflect the attitudes of all U.S. medical students. [25] Second, the physician survey was distributed in mid-2012, approximately 30 months before the medical student survey, which was distributed in early 2015. Had physicians and students been surveyed at the same time, their responses may have been different. Third, this cross-sectional study describes physician and student attitudes at one point in time. Longitudinal studies are needed to determine if the attitudes of either group change over time (e.g., if students’ positive attitudes toward cost-conscious care are sustained during residency training). Finally, the U.S. healthcare environment is changing rapidly (e.g., with the signing of the Affordable Care Act into law in March, 2010 and subsequent implementation, modification, or repeal of its various provisions). These changes may influence the attitudes of both physicians and students. Thus, attitudes should not be presumed static, and specific inferences from cross-sectional data from two different datasets should be treated with caution.

## Conclusions

These limitations notwithstanding, this study demonstrates that most U.S. practicing physicians and medical students believe physicians have a responsibility to reduce healthcare costs and limit unnecessary testing while also protecting and promoting the wellbeing of individual patients. However, medical students are more accepting of cost-conscious care, even when compared to the practicing physicians most proximate to them in age. This may be due to the combined effects of generational differences, new medical school curricula, students’ relative inexperience providing cost-conscious care within complex healthcare systems, and the rapidly evolving U.S. healthcare environment. Medical students may thus have the potential to serve as positive change agents in their learning environment with respect to cost-conscious care, and medical educators should ensure that students are equipped with the knowledge, skills, and support they need to play this important role.

## Abbreviations

AMA: American Medical Association
U.S.: United States
